# mRNA vaccines: A novel weapon to control infectious diseases

**DOI:** 10.3389/fmicb.2022.1008684

**Published:** 2022-10-04

**Authors:** Yuying Tian, Zhuoya Deng, Penghui Yang

**Affiliations:** ^1^Faculty of Hepato-Pancreato-Biliary Surgery, Institute of Hepatobiliary Surgery, The First Medical Center, Chinese PLA General Hospital, Beijing, China; ^2^Inner Mongolia Medical University, Hohhot, China

**Keywords:** mRNA vaccine, infectious disease, COVID-19, influenza virus, delivery

## Abstract

Infectious diseases have always threatened human life, but with the development of vaccines, effective strategies for preventing and controlling these diseases have become available. The global outbreak of COVID-19 ushered in the advent of mRNA vaccine technologies, which quickly led to the introduction of mRNA vaccines effective against SARS-CoV-2. The success of this approach has stimulated research into the use of mRNA vaccines in the fight against other emerging as well as remerging infectious diseases. This review examines the constructive strategies and delivery systems used in mRNA vaccines and provides an overview of current clinical trials of those vaccines in the prevention of infectious diseases. The underlying mechanisms of mRNA vaccines are also discussed, including the double-edged sword of the innate immune response. Finally, the challenges but also the potential of mRNA vaccines are considered.

## Introduction

According to the World Health Statistics Report published by the World Health Organization in 2022, infectious and communicable diseases kill millions of people every year (WHO, 2022). For example, the COVID-19 pandemic that began in late 2019 currently remains a threat. As of May 2022, the WHO reported that the number of confirmed COVID-19 cases worldwide exceeds 153 million, with more than 3.2 million deaths (WHO, 2022).

Since the introduction of the cowpox vaccine in 1796, humans have been able to prevent ~ 30 diseases through vaccination (Standaert and Rappuoli, 2017). The main vaccines available worldwide are those based on inactivated or attenuated pathogens, recombinant protein vaccines, and nucleic acid vaccines. However, these approaches have not led to vaccines against tuberculosis, malaria, and acquired immunodeficiency syndrome (AIDS). Moreover, due to the long production cycle of traditional vaccines, it is not possible to respond quickly to the emergence of major infectious diseases or to produce vaccines able to induce strong antibody and immune responses.

**Figure 1:**
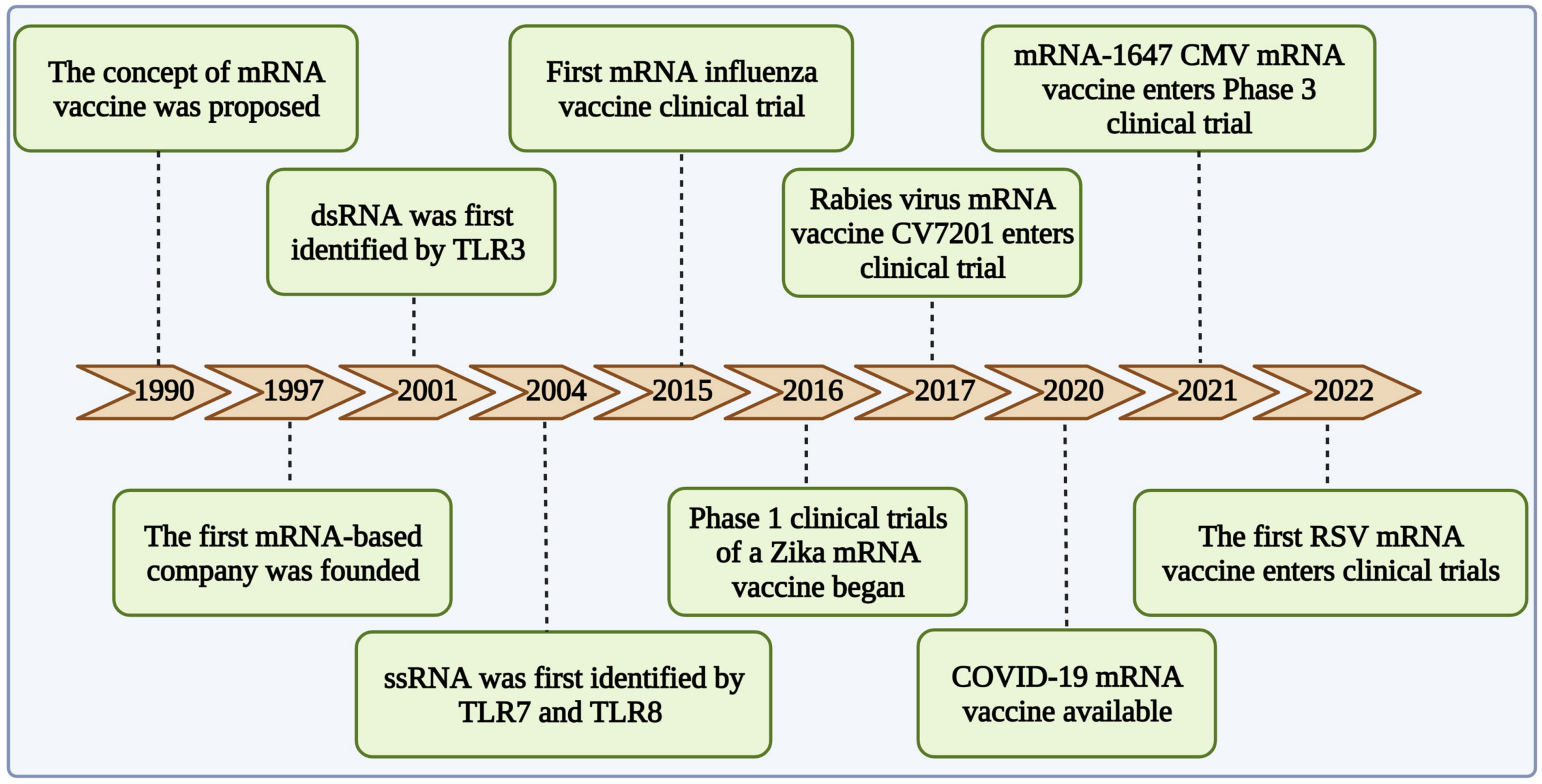
Development of mRNA vaccine in infectious diseases.

mRNA vaccines were first proposed by Wolff et al. (1990), they have been under development for the last 30 years (Figure 1). With recent advances, they are safe, highly effective, and more easily adaptable than conventional vaccines. Moreover, their cell-free production allows them to be produced more rapidly than inactivated vaccines. Unlike viral or DNA vaccines, mRNA vaccines are not integrated into the host genome (Zhang et al., 2020b). Instead, mRNA vaccines are designed to rapidly express the encoded antigen in the body and thereby quickly elicit an immune response (Tusup et al., 2021). Although the instability of single-stranded mRNA and the inefficiency of *in vivo* delivery were initially challenging (Asrani et al., 2018), these problems were eventually solved. Optimization of the mRNA sequence scheme (Haabeth et al., 2021), the development of more efficient delivery vectors (Zhao et al., 2021), and the control of the inflammatory response induced by exogenous mRNA enabled the wider application of the technology (Linares-Fernández et al., 2020a).

## Sequence construction and delivery optimization of mRNA vaccines

**Figure 2:**
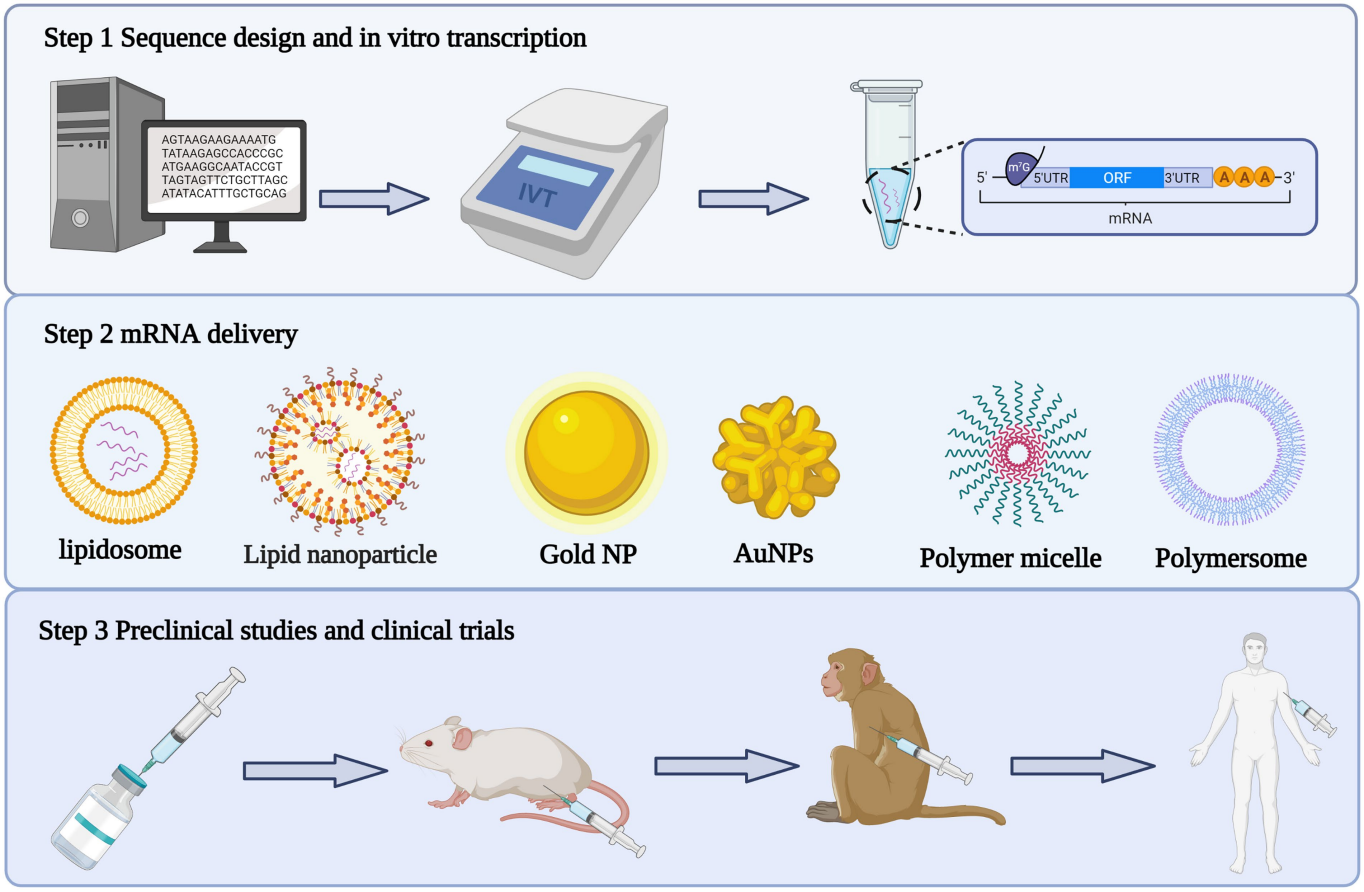
Production process of mRNA vaccine. The first step is to design the antigen sequence, construct the plasmid vector, linearize the plasmid and synthesize mRNA through *in vitro* transcription. The second step is to package the mRNA transcribed *in vitro* with appropriate delivery system. The third step is to carry out preclinical and clinical trials of the encapsulated mRNA complex.

The unstable nature of mRNA was addressed by optimizing both the sequence of the mRNA template *in vitro* and the mRNA delivery system. Not only did this improve the translation efficiency of the exogenous mRNA, it also reduced its immunogenicity (Figure 2).

### Sequence optimization of mRNA vaccines

Eukaryotic mRNA consists of a 5′ cap, a 5′ untranslated region (UTR), an open reading frame (ORF), a 3′ UTR, and a poly(A) tail (Cai et al., 2021). mRNA vaccine sequences also need to contain these elements. In eukaryotic organisms, mRNA is transcribed in the nucleus using DNA as a template and ATP, UTP, GTP, and CTP as raw materials, in accordance with the principle of complementary base pairing and by the action of RNA polymerase (Wadhwa et al., 2020). The mRNA in mRNA vaccines is transcribed *in vitro*(Li et al., 2018). *In vitro* transcription (IVT) begins with an ORF encoding the target protein and a suitable UTR, followed by the addition of the 5′ cap and poly(A) tail (Cai et al., 2021).

The eukaryotic 5′ cap is composed of 7-methylguanosine (M7G) linked to the mRNA *via* a 5′-5′-triphosphate bridge (PPP; M7GpppN; Ramanathan et al., 2016). It is an essential element in the translation of mRNA into protein. The 5′ cap structure mediates the binding of eukaryotic translation initiation factor 4E (ElF4E) while protecting the mRNA from degradation by the nucleic acid exonuclease Xrn1p, the activity of which proceeds from 5′ to 3′ (Masison et al., 1995). The 5′ cap is also required for mRNA transcribed *in vitro*, to prevent its degradation and enhance the stability of translation. Initially, the cap was sealed by an enzymatic capping reaction (Kwon et al., 2018a), but this approach was cumbersome and several cap analogs were developed, such as the formation of excess capped dinucleotides (e.g., m7GpppG) with natural guanosine triphosphate (GTP) during IVT (Jemielity et al., 2003). However, the cap analog can be inserted into the end of the mRNA in either a forward or reverse direction, which in the latter case would prevent the initiation of translation (Pasquinelli et al., 1995). The subsequent development of an anti-reverse cap analog (ARCA:3′-O-Me-m7G(5′)ppp(5′)G) solved the problem of reverse insertion (Stepinski et al., 2001) and improved the mRNA translation efficiency. Among the more widely used IVT systems currently in use are anti-inverse cap analogs as well as the bovine pox plus capase system.

UTRs, located on either side of the ORF, are key sequences containing specific motifs that bind to regulatory proteins to regulate translation (Sweeney et al., 1996). Therefore, precise regulation of target gene expression can be achieved by modifying the UTRs. For example, mRNA-2416, an mRNA tumor vaccine developed by Moderna, has an miR-122 sequence inserted in the 3′ UTR that prevents the translated antigen from acting in normal liver tissue (Febbraio et al., 2019). The UTR sequence also affects the efficiency of mRNA translation. For example, alpha- and beta(beta)-bead protein UTRs are more stable and improve translation efficiency; they are thus among the non-coding regions most commonly used by researchers (Kreiter et al., 2010a; Pardi et al., 2013; Chakraborty et al., 2021). In addition to bead protein UTRs, human heat shock protein UTRs and several viral UTRs are used to enhance protein expression(Kreiter et al., 2010a). The length and secondary structure of the UTRs are also important, as they influence the migration of the 40s ribosomal subunit bound to the mRNA. Therefore, UTRs should not contain sequences that form secondary structures, nor should they be too long (Kwon et al., 2018a).

The ORF is the core sequence of the mRNA vaccine and it serves as a template for the translation of the target protein. The base composition of the ORF is also a determinant of the translational activity and stability of the mRNA. Thus, reducing the frequency of UU and UA dinucleotides in the ORF protects the mRNA against degradation by endonucleases (Al-Saif and Khabar, 2012). Furthermore, codons should be optimized to improve translation efficiency, such as by replacing rarely used with more frequently used codons based on host tRNA preferences and by removing RNA secondary structure, potential splice motifs, and other sequences that may interfere with efficient mRNA processing (Xu et al., 2020). Nonetheless, not all mRNAs that are translated efficiently express the correct protein product. Some proteins need to be translated slowly, as they include rare bases and their folding and modification are complex (Kimchi-Sarfaty et al., 2007). Mammalian codons usually have a G or C in the third, degenerate position, and such sequences are more efficiently expressed than those whose codons end in A or T (Zhong et al., 2005).

The poly(A) tail also regulates mRNA stability and translation efficiency, including by acting in concert with the 5′ cap, the internal ribosome entry site, and other determinants (Passmore and Coller, 2022). There are two ways of tailing *in vitro* transcribed mRNA, one is to add poly(T) to the IVT template and the other is to perform IVT to obtain the initial product and then perform the entire tailing process in a two-step reaction by enzymatically extending the IVT mRNA using recombinant poly(A) polymerase. The advantage of enzymatic polyadenylation is the ability to add a poly(A) tail of 100–200 nucleotides (nt), depending on the length of the reaction. However, there are limitations to the enzymatic addition of poly(A) tails. Enzymatic tail dragging does not allow precise control of the amount of A-tail added, and for eukaryotes the optimum tail length is 120–150 nt (Sahin et al., 2014). Therefore, in clinical applications, tail addition is generally performed directly in the template, as it allows precise control of the length of the poly(A) tail.

*In vitro* transcription generates byproducts, such as double-stranded RNA, that are recognized by pattern recognition receptors (PRRs) in the cell, triggering an innate immune response and inducing the production of type I interferon (IFN), which may inhibit protein synthesis or lead to cell death, thereby affecting the therapeutic efficacy of the mRNA (discussed in “Delivery of mRNA vaccines”; Mu and Hur, 2021). Therefore, either the generation of those by-products must be reduced or the desired IVT product must be purified. mRNAs that include modified nucleotides can be recognized by the cell, such that translation efficiency is improved (Anderson et al., 2011) while the negative effects of autoimmunogenicity on mRNA vaccines are avoided (Karikó et al., 2005). Common modified nucleotides are pseudouridine (ψ), N6-methyladenosine (m6A), 5-methylcytidine (m5C),2-thiouridine (s2U) and N1-methylpseudouridine (m1ψ). Their use in IVT changes the type of RNA product synthesized, by altering the behavior of the RNA polymerase. For example, the replacement of original by modified nucleotides in IVT reduced antisense-mediated double-stranded (ds) RNA by-products when ψ, M1ψ, or M5C, but not M6A was used(Mu et al., 2018). In addition, translation efficiency can be improved and immunogenicity reduced by the physical removal of dsRNA. Standard purification methods, including LiCl, alcohol precipitation, size exclusion and ion exchange chromatography, as well as silica-matrix-based purification methods are not effective for removing dsRNA impurities from IVT mRNA (Baiersdörfer et al., 2019b). Instead, ion-pair reversed-phase high-performance liquid chromatography is the most effective method to eliminate dsRNA contamination of long IVT mRNAs. However, this method has not been universally adopted due to the high toxicity and the high cost of the eluent (Weissman et al., 2013). BioNTech therefore developed a purification strategy based on the selective binding of dsRNA to cellulose in ethanol-containing solutions of IVT mRNA products (including dsRNA) to obtain IVT mRNA with high purity (Baiersdörfer et al., 2019a).

### Delivery of mRNA vaccines

Mitigation of both the effects of mRNA instability and the difficulty of mRNA passage through cell membranes can be achieved through the use of a suitable carrier able to deliver the encapsulated post-IVT mRNA. The development of efficient and safe mRNA vaccine delivery systems has been critical to successful mRNA vaccines. During the development of a SARS-CoV-2 vaccine, new delivery methods were introduced, among which the most widely used are lipid nanoparticles (LNPs). In fact, the mRNA vaccines developed by Moderna and BioNTech are currently encapsulated in LNPs.

LNPs are a mixture of cholesterol, ionizable lipids, PEG lipids and co-lipids in various proportions (Beck et al., 2021; Miao et al., 2021). BioNTech introduced BNT162b2, an LNP-formulated nucleoside-modified mRNA vaccine that confers 95% protection against COVID-19, with only mild-to-moderate pain at the injection site (Skowronski and De Serres, 2021a). The Moderna vaccine mRNA-1273 also uses LNP encapsulation and has a similarly high prophylactic effect (Baden et al., 2021). A more classical delivery method makes use of the protamine complex. CureVac’s RNActive^©^ technology is a mixture of mRNA and this fish sperm protein (Carralot et al., 2004; Kallen et al., 2013a). The company then applied this technology to vaccines for other infectious diseases. Their mRNA vaccine encoding A/PoRico/8/1934 (PR8HA), when given to mice in two doses 3 weeks apart, produced effective neutralizing antibodies and long-lasting immune protection, regardless of age (Kallen et al., 2013a). The vaccine can be stored at −20°C, and at 37°C has the same protective effect in mice (Petsch et al., 2012).

Other novel delivery strategies introduced during the COVID-19 pandemic and with the broader aim of overcoming the limitations of existing delivery systems included self-assembled polymeric micelle delivery systems based on polyethyleneimine modified with vitamin E succinate (Ren et al., 2021a). A PVES/mRNA vaccine encoding the SARS-CoV-2 RBD antigen was shown to induce a Th1-type immune response, with no inflammatory reaction at the injection site and no significant lesions in other vital organs (Ren et al., 2021a). The nanohydrogel system developed by Wang et al. consists of an X-shaped DNA scaffold and DNA linker that compresses the nanohydrogel into a ball, which then enters the cell through cytokinesis and uses a pH change as a switch to control mRNA release (Fu et al., 2021). Londiwe et al. developed a delivery system based on folic-acid-modified, poly amidoamine-generation-5 (PAMAM G5D)-grafted gold nanoparticles (AuNPs; Mbatha et al., 2021a). Both delivery and translation efficiency were significantly better than achieved with AuNPs alone (Mbatha et al., 2021a). AuNPs act as adjuvants to enhance the immune response to vaccines. They are biocompatible, inexpensive, and safe to produce. Other inorganic nanomaterials used for the delivery of nucleic acids include iron oxide nanoparticles, mesoporous silica nanoparticles, and calcium phosphate nanoparticles (Liu et al., 2021).

## Clinical application of mRNA vaccines in infectious diseases

In 1993, mRNA encoding influenza nucleoprotein delivered by a liposome package was injected into mice and induced virus-specific T-cell responses. This was the first demonstration of the use of an mRNA vaccine to treat an infectious disease. Compared to traditional vaccines for infectious diseases, such as inactivated attenuated vaccines, subunit vaccines, and protein vaccines, mRNA vaccines have outstanding advantages. However, at the time, their rapid degradation and the instability of single-stranded mRNA hindered their development for clinical use. With the advent of methods to optimize the mRNA sequence together with improved IVT and packaging technologies, the use of mRNA vaccines to treat infectious diseases in humans has become possible. In addition to the mRNA vaccines introduced in response to the COVID-19 pandemic (discussed below), mRNA vaccines for the treatment of influenza, respiratory syncytial virus, HIV, Zika virus (ZIKV), and other infectious diseases have passed the preclinical trial stage and are now being tested in clinical trials.

### Clinical application of mRNA vaccine in COVID-19

In late 2019, a novel coronavirus, SARS-CoV-2 was detected and isolated in China. The disease caused by the virus is referred to as COVID-19. As SARS-CoV-2 continues to mutate and the rate of infection remains high, research focused on the development of effective drugs and vaccines continues at a high pace. As of April 27, 2022, at least 28 mRNA vaccines for COVID-19 had entered clinical trials (Table 1), and five had entered phase 3 clinical trials, with 3 now in phase 4. Moderna’s mRNA-1273 /Spikevax and BNT162b2 were approved for use by the U.S. Food and Drug Administration. Another 24 mRNA vaccines are under preclinical study (Table 2).

**Table 1:** Clinical application of COVID-19 mRNA vaccine (from https://www.who.int/publications/m/item/draft-landscape-of-covid-19-candidate-vaccines, 30 August 2022).

Vaccine platform description	Type of candidate vaccine	Number of doses	Route	Developers	Phase	Number
RNA based	mRNA-1273	2	IM	Moderna + National Institute of Allergy and Infectious Diseases (NIAID)	Phase 4	NCT04760132
RNA based	BNT162b2 (3 LNP-mRNAs), also known as "Comirnaty"	2	IM	Pfizer/BioNTech + Fosun Pharma	Phase 4	NCT04760132
RNA based	CVnCoV Vaccine	2	IM	CureVac AG	Phase 3	NCT04674189
RNA based	ARCT-021	NR	IM	Arcturus Therapeutics	Phase 2	NCT04668339
RNA based	LNP-nCoVsaRNA	2	IM	Imperial College London	Phase 1	ISRCTN17072692
RNA based	SARS-CoV-2 mRNA vaccine (ARCoV)	2	IM	Academy of Military Science (AMS), Walvax Biotechnology and Suzhou Abogen Biosciences	Phase 3	NCT04847102
RNA based	ChulaCov19 mRNA vaccine	2	IM	Chulalongkorn University	Phase 1/2	NCT05231369
RNA based	PTX-COVID19-B, mRNA vaccine	2	IM	Providence Therapeutics	Phase 2	NCT05175742
RNA based	CoV2 SAM (LNP) vaccine. A self-amplifying mRNA (SAM) lipid nanoparticle (LNP) platform + Spike antigen	2	IM	GlaxoSmithKline	Phase 1	NCT04758962
RNA based	mRNA-1273.351.	3	IM	Moderna + National Institute of Allergy and Infectious Diseases (NIAID)	Phase 4	EUCTR2021-000930-32
RNA based	MRT5500, an mRNA vaccine candidate	2	IM	Sanofi Pasteur and Translate Bio	Phase 2	NCT04798027
RNA based	DS-5670a, coronavirus-modified uridine RNA vaccine (SARS-CoV-2)	2	IM	Daiichi Sankyo Co., Ltd.	Phase 2/3	JPRN-jRCT2071210106
RNA based	HDT-301: Self-replicating mRNA vaccine formulated as a lipid nanoparticle.	2	IM	SENAI CIMATEC	Phase 1	NCT04844268
RNA based	mRNA-1283	2	IM	ModernaTX, Inc.	Phase 1	NCT04813796
RNA based	EXG-5003; a temperature-sensitive self-replicating RNA vaccine expressing the receptor binding domain of the SARS-CoV-2 spike protein.	1	ID	Elixirgen Therapeutics, Inc	Phase 1/2	NCT04863131
RNA based	mRNA COVID-19 vaccine	2	IM	Shanghai East Hospital and Stemirna Therapeutics	Phase 1	ChiCTR2100045984
RNA based	LNP-nCOV saRNA-02 vaccine; Self-amplifying RNA (saRNA) encapsulated in lipid nanoparticles (LNP)	2	IM	MRC/UVRI and LSHTM Uganda Research Unit	Phase 1	NCT04934111
RNA based	mRNA-1273.211. A multivalent booster candidate combining mRNA-1273 plus mRNA-1273.351.	1	IM	ModernaTX, Inc.	Phase 2/3	NCT04927065
RNA based	ARCT-154 mRNA Vaccine	2	IM	Arcturus Therapeutics, Inc.	Phase 3	ISRCTN15779782
RNA based	ARCT-165 mRNA Vaccine	2	IM	Arcturus Therapeutics, Inc.	Phase 1/2	NCT05037097
RNA based	ARCT-021 mRNA Vaccine	2	IM	Arcturus Therapeutics, Inc.	Phase 1/2	NCT05037097
RNA based	HDT-301 vaccine	1-2	IM	HDT Bio	Phase 1	NCT05132907
RNA based	VLPCOV-01, self-amplifying RNA vaccine against the coronavirus	2	IM	VLP Therapeutics Japan GK	Phase 1	jRCT2071210067
RNA based	EG-COVID vaccine	3	IM	EyeGene Inc.	Phase 1/2	NCT05188469
RNA based	Coronavirus mRNA vaccine (LVRNA009)	2	IM	AIM Vaccine and Liverna Therapeutics	Phase 1	ChiCTR2100049349
RNA based	mRNA-1273.529 - Booster	1	IM	ModernaTX, Inc.	Phase 2/3	NCT05249829
RNA based	CV2CoV, mRNA vaccine	1	IM	CureVac AG	Phase 1	NCT05260437
RNA based	mRNA vaccine (MIPSCo-mRNA-RBD-1)	1	IM	University of Melbourne	Phase 1	NCT05272605
RNA based	COVID-19 mRNA Vaccine (SYS6006)	2	IM	CSPC ZhongQi Pharmaceutical Technology Co., Ltd.	Phase 2	NCT05439824
RNA based	mRNA GEMCOVAC-19 (COVID-19 vaccine)	2	IM	Gennova Biopharmaceuticals Limited	Phase 2/3	CTRI/2022/04/041880
RNA based	Lyophilized COVID-19 mRNA Vaccine	1	IM	Wuhan Recogen Biotechnology Co., Ltd.	Phase 1	NCT05366296
RNA based	mRNA vaccine (Adenovirus Type 5 Vector)	2	IM	CanSino Biologics Inc.	Phase 3	NCT05442684
RNA based	A self-amplifying RNA (saRNA) boost vaccine (AAHI-SC2 and AAHI-SC3)	1	IM	ImmunityBio, Inc.	Phase 1/2	NCT05370040
RNA based	RQ3013: SARS-CoV-2 mRNA Chimera Vaccine	1	IM	Walvax Biotechnology; Shanghai RNACure Biopharma	Phase 1	NCT05396573
RNA based	mRNA-1273.214 (Booster)	2	IM	ModernaTX	Phase 3	NCT05436834
RNA based	mRNA-1073; (COVID-19/Influenza) Vaccine	2	IM	ModernaTX	Phase 1/2	NCT05375838
RNA based	RVM-V001	1	IM	RVAC Medicines	Phase 1	NCT05420077
RNA based	ABO1009-DP (COVID-19 Omicron) mRNA Vaccine	1	IM	Suzhou Abogen Biosciences Co., Ltd.	Phase 1	NCT05433194
RNA based	Self-Amplifying Messenger Ribonucleic Acid (samRNA) Vaccines	2	IM	Gritstone bio, Inc.	Phase 1	NCT05435027
RNA based	Investigational CV0501 mRNA COVID-19 Vaccine	1	IM	GlaxoSmithKline	Phase 1	NCT05477186

**Table 2:** Preclinical study of A COVID-19 mRNA vaccine (from https://www.who.int/publications/m/item/draft-landscape-of-covid-19-candidate-vaccines, 30 August 2022).

Vaccine platform description	Type of candidate vaccine	Coronavirus target	Same platform for non-Coronavirus candidates	Developers
RNA based	saRNA formulated in a NLC	SARS-CoV2		Infectious Disease Research Institute/ Amyris, Inc.
RNA based	LNP-encapsulated mRNA encoding S	SARS-CoV2		Max-Planck-Institute of Colloids and Interfaces
RNA based	Self-amplifying RNA	SARS-CoV2		Gennova
RNA based	mRNA	SARS-CoV2		Selcuk University
RNA based	LNP-mRNA	SARS-CoV2		Translate Bio/Sanofi Pasteur
RNA based	LNP-mRNA	SARS-CoV2		CanSino Biologics/Precision NanoSystems
RNA based	LNP-encapsulated mRNA cocktail encoding VLP	SARS-CoV2		Fudan University/ Shanghai JiaoTong University/RNACure Biopharma
RNA based	LNP-encapsulated mRNA encoding RBD	SARS-CoV2		Fudan University/ Shanghai JiaoTong University/RNACure Biopharma
RNA based	Replicating Defective SARS-CoV-2 derived RNAs	SARS-CoV2		Centro Nacional Biotecnología (CNB-CSIC), Spain
RNA based	LNP-encapsulated mRNA	SARS-CoV2	MERS	University of Tokyo/ Daiichi-Sankyo
RNA based	Liposome-encapsulated mRNA	SARS-CoV2		BIOCAD
RNA based	Several mRNA candidates	SARS-CoV2		RNAimmune, Inc.
RNA based	mRNA	SARS-CoV2		FBRI SRC VB VECTOR, Rospotrebnadzor, Koltsovo
RNA based	mRNA	SARS-CoV2		China CDC/Tongji University/Stermina
RNA based	mRNA in an intranasal delivery system	SARS-CoV2		eTheRNA
RNA based	mRNA	SARS-CoV2		Greenlight Biosciences
RNA based	mRNA	SARS-CoV2		IDIBAPS-Hospital Clinic, Spain
RNA based	mRNA	SARS-CoV2		Providence Therapeutics
RNA based	mRNA	SARS-CoV2		Cell Tech Pharmed
RNA based	mRNA	SARS-CoV2		ReNAP Co.
RNA based	D614G variant LNP-encapsulated mRNA	SARS-CoV2		Globe Biotech Ltd
RNA based	Encapsulated mRNA	SARS-CoV2		CEA
RNA based	Recombinant, prefusion stabilized SARS-CoV-2 Spike antigen	SARS-CoV2		Medigen Vaccines Biologics Corp (MVC)/Vaxess Technologies (MIMIX)
RNA based	ZIP1642 is a self-amplifying RNA vaccine encapsulated in an LNP, which encodes for multiple antigens, including the Spike (S) protein.	SARS-CoV2		Ziphius Vaccines and Ghent University
RNA based	LNP-mRNA	SARS-CoV2	Multiple candidates	Certest Biotec

#### BNT162b1 and BNT162b2

At the beginning of the COVID-19 outbreak, Pfizer and BioNTech collaborated to develop four mRNA vaccines. After screening, two were selected as candidates for clinical trials: BNT162b1 encodes the receptor binding domain of SARS-COV-2 and adds the T4 fibrin folding domain for trimerization to increase immunogenicity. BNT162b2 encodes the full-length spike protein of SARS-CoV-2 that has been modified to ensure its conformational stability (Vogel et al., 2020).

Both vaccines were tested in preclinical trials in mice and rhesus monkeys. In mice, intramuscular injections of either vaccine induced a dose-dependent antibody response. In rhesus monkeys, serum antibody titers were detected 7 days after the administration of two doses of either vaccine and were 18 times higher than those induced during recovery from viral infection. After vaccination and subsequent SARS-CoV-2 challenge, viral RNA was not detected in lung lavage fluid and a histopathological examination found no evidence of obvious lung lesions (Teo, 2021).

Then BNT162b1 and BNT162b2 were tested in phase 1 clinical trials in healthy adults 18–55 years of age, both in the United States and in Germany. Individuals in the low-dose group given a second dose of the vaccine had titers of serum neutralizing antibody 4.6 times higher than those of convalescing patients and the response was dose-dependent (Mulligan et al., 2020). An immunological analysis showed that both vaccines induced Th1 cell responses(Sahin et al., 2020). While the number of adverse events in response to BNT162b1 also increased with the dose, BNT162b2 recipients had fewer systemic adverse reactions. An age-extended trial was subsequently conducted in the United States to evaluate the immunogenicity and safety of two doses of BNT162b1 or BNT162b2 in adults 65–88 years of age. The results showed no significant difference in immunogenicity between the two vaccines, but, again, BNT162b2 was better tolerated (Payne et al., 2021). Thus, in the subsequent phase 1 trial only BNT162b2 was evaluated.

The phase 3 clinical study to determine the safety and effectiveness of the vaccine was conducted at 152 sites worldwide, including in the United States, Germany, Brazil, Argentina, Turkey, and South Africa. Study participants were 16 years of age and older and received two doses of either placebo or 30 μg BNT162b2. The 37,706 recipients of the second vaccine dose were free of infection during the first 2 months; eight vaccine recipients contracted COVID-19 compared to 162 who received the placebo. The study results showed that BNT162b2 has a good protective effect in different age groups, races, and regions. Adverse reactions were mainly local pain, headache, and fatigue. The incidence of serious adverse events was low, with no significant difference between the vaccine and placebo groups (Skowronski and De Serres, 2021a).

#### mRNA-1273

Following the release of the SARS-CoV-2 genome, Moderna developed an mRNA vaccine, mRNA-1273 (Corbett et al., 2020), delivered by LNPs. The mRNA sequence encodes the spike protein stably expressed on SARS-CoV-2. To produce a perfused stable SARS-Cov2 protein, mutations were substituted into residues 986 and 987 of the protein.

Preclinical data showed that mice inoculated with two doses of mRNA-1273 produced a strong neutralizing antibody response. No SARS-CoV-2 infection occurred in mice vaccinated within 3 months prior to the challenge. The protective efficacy of the vaccine was dose-dependent. In non-human primates inoculated with mRNA-1273, neutralizing antibody titers were higher than those in the serum of humans recovering from infection and the immune response was Th1 biased (Wrapp et al., 2020).

In a phase 1 dose escalation and age extension clinical trial, dose-dependent antibody responses were observed in both young and elderly populations after two doses of vaccine. There were mild adverse reactions, but no major adverse reactions. However, in the high-dose group (250 μg), 21% had serious adverse reactions after the second dose. With increasing time after inoculation, the levels of neutralizing and binding antibodies decreased, but remained high (Jackson et al., 2020).

The phase 3 trial evaluated the broad protective efficacy and safety of mRNA-1273 at a dose of 100 μg. Moderna enrolled 30,000 people over the age of 18 to receive two doses of either placebo or vaccine. The participants included the elderly and patients with chronic diseases. The protective efficacy of the vaccine was assessed at 94.5%. Severe SARS-CoV-2 infection occurred in 11 patients in the placebo group and none in the vaccine group(Anderson et al., 2020).

#### ARCoV and SW0123

Abogenbio developed an mRNA vaccine (ARCoV) that encodes the RBD spike protein of SARS-CoV-2 and is delivered with LNPs. Preclinical studies of the vaccine showed that it induces neutralizing antibodies and Th1-biased immune responses in mice and non-primates, and protects against SARS-CoV-2 infection. In subsequent phase 1 trials, most participants received two doses of vaccine, with neutralizing antibodies peaking 14–28 days after the second dose and the highest antibody titer induced by the 15-μg dose. There were only mild adverse events, such as fever, and no serious adverse events. The incidence of adverse events was similar between the first and second vaccinations. Large population safety and protective evaluations are being conducted (Chen et al., 2022).

SW0123, developed by stamina, encodes the full-length SARS-CoV-2 spike protein and is delivered using lipoprotein particles. Studies in mice and non-human primates showed that SW0123 induces high titers of neutralizing antibodies and a Th1-biased T-cell response. It has good protective effects against SARS-CoV-2 and its D614G and N501Y variants (Zhang et al., 2020a). After intramuscular injection, SW0123 was not significantly enriched in the liver or other important organs. Then SW0123 was entered into phase 1 clinical trials in China (Yang et al., 2021).

### Application of mRNA vaccines in influenza

An estimated 7.9 million people in the United States contracted influenza from October 2021 to April 2022, and about 8,200 people died from the disease during that time. Influenza vaccination is a powerful measure to prevent infection with influenza viruses. WHO, in collaboration with a number of global agencies, provides semi-annual recommendations on seasonal influenza vaccine combinations expected to be effective for the northern and southern hemispheres, based on data from the Global Influenza Detection and Response System, responsible for influenza detection around the world. According to the CDC (CDC Seasonal Flu Vaccine Effectiveness Studies, 2021-2022), from 2004 to 2019, the effectiveness of the flu vaccine ranged from 10 to 60%, which reflects the fact that the virus is prone to mutation and vaccines based on predictions are often not fully protective. Once a new variant of influenza emerges and triggers a pandemic, it is very difficult for traditional influenza vaccines to be developed, preclinically tested, and then produced in large quantities fast enough to meet global needs (Watson and Pebody, 2011). These obstacles can be overcome using mRNA vaccines.

After the sequences of the HA and neuraminidase (NA) genes of the H7N9 influenza virus in China were published on the data sharing network, researchers generated candidate vaccines within 8 days, through a combination of cell-free gene synthesis and self-amplifying mRNA (SAM) vaccine technology. Mice were immunized twice, with both immunizations producing neutralizing antibodies in amounts sufficient to resist viral attack (Hekele et al., 2013). mRNA vaccines can be altered to rapidly respond to pandemic-scale influenza, allowing a broader spectrum of universal influenza vaccines to be developed. In Freyn et al. (2020), mice were intradermally injected with a set of conserved influenza virus antigens (hemagglutinin stem, neuraminidase, stroma-2 ion channel, and nucleoproteins), which induced a strong immune response. After a single immunization, the nucleoside-modified mRNA-LNP vaccine provided immunological protection against 500 times the lethal dose of H1N1 virus, while the combination vaccine protected at a dose of 50 ng per antigen. The broad protective potential of the single-dose combination vaccine against influenza A viruses was also reported. Zhuang et al. (2020) constructed an mRNA vaccine against the H1N1 influenza virus and immunized C57BL/6 mice *via* intranasal administration. The vaccine triggered both humoral and cellular immune responses and completely protected the mice against H1N1 at tenfold the lethal dose. Chivukula et al. (2021) developed Cal09 HA/Sing16 HA, Sing16 NA, Mich15 NA, and Perth09 NA as monovalent or polyvalent vaccines. Both HA and NA mRNA-LNP vaccines were shown to induce antigen-specific antibodies and immune responses in non-human primates and to protect mice from viral attack.

The three major mRNA vaccine manufacturers have also contributed to the flu vaccine pipeline. For example, a trial of Moderna’s first seasonal influenza mRNA vaccine, mRNA-1010-P101, has completed phase 2 clinical recruitment and is in preparation for phase 3 clinical trials. The vaccine encodes WHO-recommended candidate strains of seasonal influenza virus, including seasonal influenza A/H1N1 and A/H3N2 as well as influenza B/Yamagata-and B/Victoria-lineages. In a phase 1 clinical trial, mRNA-1,010 successfully enhanced geometric mean titers in hemagglutination inhibition measurements for all strains, at all doses tested, and in both young and elderly adults at 29 days after vaccination. The geometric mean fold rise (GMFR) of influenza A strains above baseline was ~ 10-fold (H1N1) and ~ 8-fold (H3N2), while that of B/Yamagata was ~ 3-fold, and that of B/Victoria ~ 2-fold. The GMFR of influenza A strains in the elderly population was ~ 6-fold (H1N1) and ~ 6-fold (H3N2), ~ 3-fold for B/Yamagata, and ~ 2 times for B/Victoria. In addition to doses of 50 and 100 μg tested in the two groups during the phase 1 trial, a small dose of 25 μg was added to the phase 2 trial. Moderna is also developing mRNA-1,011, mRNA-1,012, MRNA-1020, and mRNA-1,030 vaccines that are not limited to seasonal influenza. Compared to mRNA-1,010, mRNA-1,011 has one more HA antigen, and mRNA-1,012 two more HA antigens. mRNA-1020 and mRNA-1030 bind NA antigens. Moderna’s four vaccines appear to be a step toward the development of a universal flu vaccine.

CVSQIV is a seasonal influenza vaccine jointly developed by CureVac and GlaxoSmithKline. Currently, no preclinical results have been published as CVSQIV is in phase I clinical recruitment status (NCT05252338). The phase 1 clinical trial evaluates safety, immunogenicity, and immunoreactivity in the 3–28 μg dose range in populations 18–55 and ≥ 65 years of age. BNT161 (mIRV) is a mRNA-monovalent influenza vaccine developed jointly by Pfizer and BioNTech that is currently in a clinical phase 1 trial (NCT05052697). The objective is to evaluate the safety and immunogenicity of a monovalent influenza vaccine (mIRV) as well as bivalent (bIRV), and quadrivalent (qIRV) influenza modRNA vaccines in adults 65–85 years of age.

At the same time, the protective effects of influenza and COVID-19 mRNA vaccines in susceptible populations are being explored in clinical trials examining the safety and immunogenicity of the two vaccines when administered successively (Izikson et al., 2022). Duke University initiated a clinical trial of simultaneous vaccination with the COVID-19 vaccine and the seasonal influenza virus quadrivalent vaccine. Recruitment has been suspended until the high flu season begins (NCT05028361). Previously, the flu vaccine that is used in conjunction with the COVID-19 vaccine is not an mRNA vaccine, as there are as yet no mature mRNA influenza vaccines. However, Moderna has begun to develop a vaccine that combined the flu vaccine with the COVID-19 vaccine, it was named mRNA-1073 (Julie, 2021). A phase 1/2 clinical trial is underway to evaluate the safety, reactivity, and immunogenicity of mrNA-1073. Two doses of mrNA-1073 were administered in one group and both mrNA-1010 and mrNA-1273 were administered in the other group to evaluate the immune response of the two inoculation methods (NCT05375838).

### Clinical application of mRNA vaccines against respiratory syncytial virus

Respiratory syncytial virus(RSV) was first identified in Blount Jr. et al. (1956). According to WHO, 64 million children worldwide are infected with RSV each year. The virus causes pneumonia that threatens not only children and the elderly but also younger adults, in whom severe respiratory symptoms may develop (Nam and Ison, 2019). Efforts to develop vaccines to prevent RSV in infants began in the 1960s. However, rather than achieving the desired results, the vaccine increased RSV infection, and two infants died (Kim et al., 1969). A formalin-inactivated RSV vaccine was subsequently tested for immunogenicity but the induced antibodies had low affinity for the epitopes (Delgado et al., 2009). A better strategy may be to subunit vaccines that retain key epitopes of RSV-F (Ngwuta et al., 2015). In 2016, the subunit vaccine developed by Novavax entered phase 2 clinical trials, but its protective efficacy was disappointing(Schmidt et al., 2012). Moderna developed a gene encoding a prefusion F glycoprotein mRNA-1345 (P101, P301) and codon optimization for RSV glycoprotein. Phase I interim clinical data in adults 65–79 years of age showed that the titer of neutralizing antibodies against RSV was 14 times higher after vaccination with mRNA-1345 vaccine than after the sham vaccine (Staff, 2022). In 2022, Moderna will test the mRNA-1345 vaccine in children, young adults, women and the elderly to evaluate tolerance, reactivity, and immunogenicity (NCT04528719).

### Clinical application of mRNA vaccine in HIV

AIDS was first identified in 1981 by Friedman-Kien (1981), and HIV was first isolated by Barré-Sinoussi et al. (1983). The United Nations established the Joint United Nations Programme on AIDS in 1996. According to its 2021 Global AIDS Progress Report, 37.7 million people worldwide were living with HIV and 680,000 died of AIDS-related diseases in 2020. Despite the large body of research on AIDS (Buchbinder et al., 2008). Treatment is limited to antiretroviral therapy and neither a vaccine to prevent the disease nor a drug to cure it has been discovered. Thus, AIDS remains an intractable medical problem. One obstacle has been that HIV can mutate quickly and integrate into the host genome, such that vaccines or drugs that target multiple HIV mutation sites are needed. mRNA vaccines offer hope because they can induce a broad range of neutralizing antibodies to HIV. Zhao et al. (2016) developed an HIV-1 gag mRNA vaccine delivered using polyethyleneimine stearic acid (PSA). Studies have shown that PSA/mRNA vaccines can be delivered into cells and effectively induce antigen-specific immune responses. Bogers et al. (2015) used cationic nanoemulsion to deliver a SAM vaccine encoding a branch of the HIV envelope glycoprotein. An evaluation of its immunogenicity and safety in rhesus monkeys showed that the vaccine induced a stronger immune response than a coated glycoprotein recombinant vaccine and had good safety. Using a mRNA vaccine co-expressing membrane-anchored HIV-1 envelope (Env) and simian immunodeficiency virus (SIV) Gag proteins, Zhang et al. (2021a) demonstrated that virus-like particles could be generated from this vaccine. As a result of rhesus macaque vaccination, broad neutralizing antibodies were produced, which reduces the risk of infection (Zhang et al., 2021a). Gómez et al. (2021) used modified 1-methyl-3′-pseudouridylyl vectors that included a T-cell multiepitope construct encoding HIV-1 Gag, Pol, and Nef proteins conserved epitopes for protection against HIV-1 infection. Valentin et al. (2022) found that LNP/mRNA vaccines expressing the HIV-1 Gag and Gag conserved region, when combined with Gag DNA vaccines, elicited strong humoral and cellular immune responses.

Over the past 20 years, several clinical trials of an AIDS mRNA vaccine have been carried out, but efficacy was achieved only at the level of humoral and cellular immune responses. For example, in a phase II clinical trial, an mRNA vaccine encoding CD40L and HIVACAT T cell immunogens delivered using dendritic cells protected against HIV infection by inducing the synthesis of an effective HIV immunogen that activated specific T-cells; however, the clinical trial was halted because of an extra start codon in the sequence (de Jong et al., 2019). Moderna’s mRNA vaccine pipeline also includes HIV vaccines, such as mRNA-1644 (NCT05001373) and mRNA-1574, both in phase 1 clinical recruitment. In preclinical studies, mrA-1644 was 79% effective in protecting against HIV infection in rhesus monkeys (Zhang et al., 2021a). In the BioNTech pipeline, HIV vaccines are still in the pre-clinical stage. Recruitment is ongoing for a NIAID-sponsored clinical trial evaluating the safety and efficacy of BG505 MD39.3, BG505 MD39.3 GP151, and BG505 MD39.3 GP151 CD4KO HIV trimer mRNA vaccines (NCT05217641).

### Clinical application of mRNA vaccine in other infectious diseases

The above clinical trials suggest that, at least in theory, the specific antigen epitope sequence of the virus can be encoded by mRNA and prepared in an mRNA vaccine. In addition to the aforementioned diseases, mRNA vaccines are being tested for use against ZIKV, rabies, and cytomegalovirus (Table 3). ZIKV is associated with fetal and placental dysfunction and birth defects during pregnancy (Brasil et al., 2016). It can be spread among humans by *Aedes* mosquitoes. During the 2016 ZIKV outbreak, Moderna worked with the Defense Advanced Research Projects Agency (DARPA) and the Biomedical Advanced Research and Development Authority (BARDA) to develop mRNA-1325-P101 over a 10-month period and demonstrated antibody production in animal studies. However, in phase 1 clinical trials, no effective antibodies were produced in humans. Moderna then developed mRNA-1893-P101 and mRNA-1893-P201, which are now in clinical trials.

**Table 3:** Clinical trials of mRNA vaccines for infectious diseases other than COVID-19.

Number	Coronavirus target	Type of candidate vaccine	Name	Route of administration	Developers	Phase	Status
NCT05252338	Seasonal Influenza	Unknown	CVSQIV	IM	CureVac AG/GlaxoSmithKline	Phase 1	Recruiting
NCT04956575	Seasonal Influenza	Influenza A (H1N1, H3N2), influenza B	mRNA-1010	IM	ModernaTX, Inc	Phase 1/2	Recruiting
NCT05333289	Seasonal Influenza	Unknown	mRNA-1030	IM	ModernaTX, Inc	Phase 1/3	Recruiting
NCT03345043	Influenza A (H7N9)	Nucleoside- modified mRNA–LNP	mRNA-1851	IM	ModernaTX, Inc	Phase 1	Completed
NCT03076385	Influenza A (H10N8)	Nucleoside- modified mRNA–LNP	mRNA-1440	IM	ModernaTX, Inc	Phase 2	Completed
NCT05330975	RSV	Nucleoside- modified mRNA–LNP	mRNA-1345	IM	ModernaTX, Inc	Phase 3	Recruiting
NCT04144348	hMPV/PIV3	Nucleoside- modified mRNA–LNP	mRNA-1653	IM	ModernaTX, Inc	Phase 1	Recruiting
NCT04917861	Zika Virus	Nucleoside- modified mRNA–LNP	mRNA-1893	IM	ModernaTX, Inc	Phase 2	Recruiting
NCT03014089	Zika Virus	Nucleoside- modified mRNA–LNP	mRNA-1325	IM	ModernaTX, Inc	Phase 1	Completed
NCT05085366	CMV	Nucleoside- modified mRNA–LNP	mRNA-1647	IM	ModernaTX, Inc	Phase 3	Recruiting
NCT03382405	CMV	Nucleoside- modified mRNA–LNP	mRNA-1443	IM	ModernaTX, Inc	Phase 1	Completed
NCT03713086	Rabies	Unmodified mRNA–LNP	CV7202	IM	CureVac AG	Phase 1	Completed
NCT03713086	Rabies	Unmodified mRNA complexed in RNActive	CV7201	IM	CureVac AG	Phase 1	Completed
NCT04062669	Rabies	Self- amplifying mRNA in cationic nanoemulsion	GSK3903133A	IM	GlaxoSmithKline	Phase 1	Active, not recruiting

Cytomegalovirus (CMV) is a herpes type II virus that causes mostly asymptomatic infections, but infections in immunocompromised patients, such as pregnant women and transplant patients (Ramanan and Razonable, 2013) can cause serious complications. CMV is also an important cause of neonatal defects (Foulon et al., 2008). A phase 2 clinical trial with the mRNA-1647 vaccine was recently completed, but the results have not yet been published, and enrollment for phase 3 clinical trials is now open. Moderna has also developed an mRNA vaccine (mRNA-1443) that is being tested in combination with mRNA-1647 for its immunogenicity and safety in healthy individuals.

Rabies virus can infect humans as well as other mammals. After an incubation period, infections in humans can cause severe neurological, potentially life-threatening symptoms (Hemachudha et al., 2013). While many vaccines against the rabies virus are available, an mRNA-based rabies vaccine (CV7201, deployed by CureVac Corp.) was first entered into clinical trials in 2017. The vaccine’s mRNA encodes a non-replicating rabies virus glycoprotein. In preclinical trials, the vaccine was effective for inducing neutralizing antibodies in mice and domestic pigs, with a larger number of CD4^+^ T cells induced than achieved with the licensed vaccine (Schnee et al., 2016). Both CV7201 and CV7202 completed phase I clinical trials and demonstrated good safety and immunogenicity (Aldrich et al., 2021). In 2020, CureVac and GSK collaborated to develop a SAM rabies virus vaccine; preclinical data showed that it was well tolerated (Stokes et al., 2020).

## Current challenges of mRNA vaccines

### Influence of exogenous mRNA immunogenicity on mRNA vaccines

**Figure 3:**
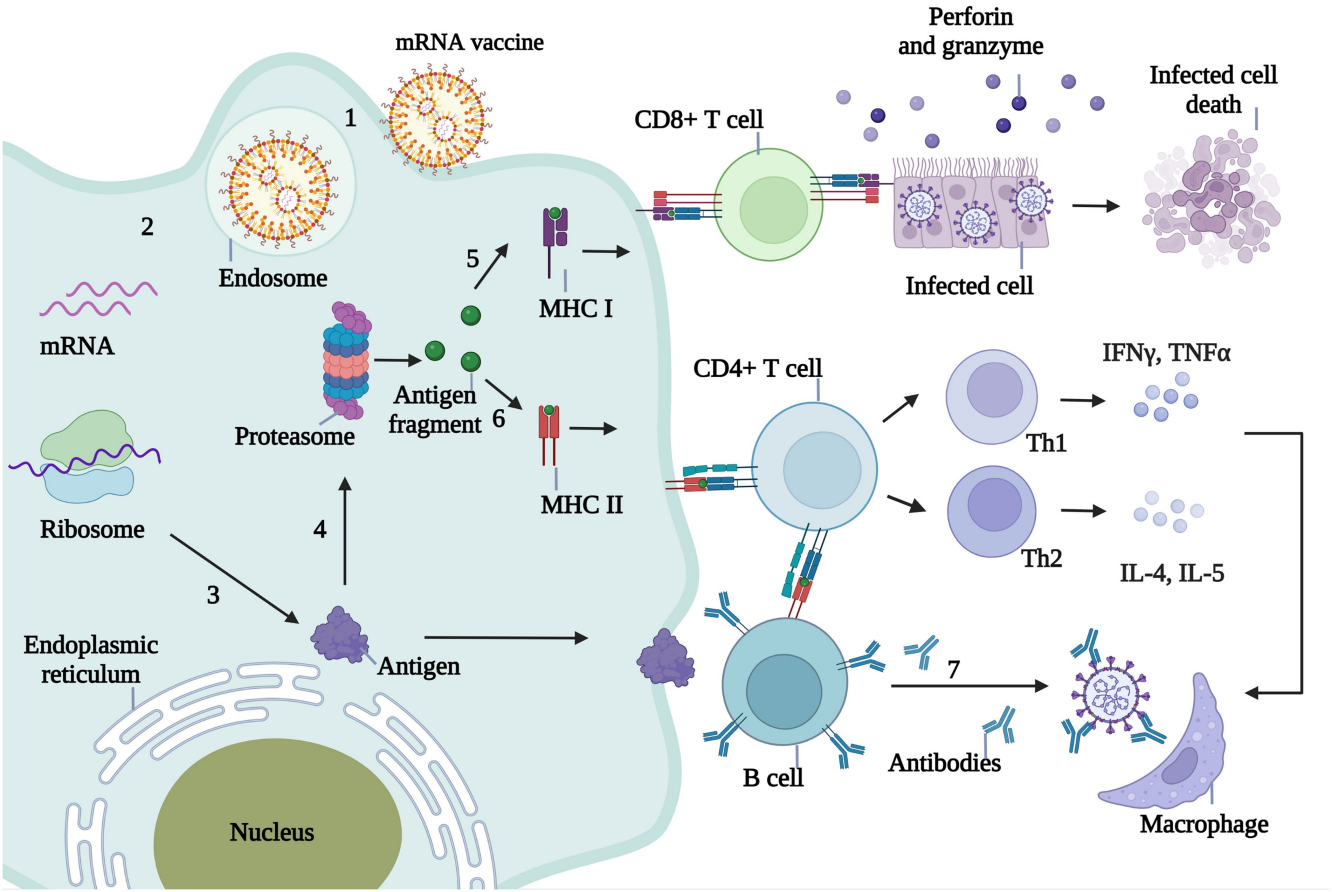
Immunological mechanism of mRNA vaccine against infectious diseases. 1. The mRNA wrapped by the delivery system enters the body and is ingested by antigen presenting cells (APC). 2. Endosomes release mRNA into the cytoplasm. 3. The mRNA in the cytoplasm is translated into the antigenic protein encoded by the ribosome. 4. Antigenic proteins in the cytoplasm are broken down into small antigenic fragments by proteasome complexes. 5. The decomposed antigen fragments were captured by MHC-I molecules and presented to CD8^+^ T cells. CD8^+^ T cells recognized the infected cells by MHC-I and released granuliase and perforin to lysate the infected cells. 6. Antigen fragments decomposed by proteasome complex can also be recognized by MHC-II molecules and presented to CD4^+^ T cells, which are divided into Th1 and Th2. Th1 cells secreted IFNγ, TNFα and Th2 cells secreted IL-4, IL-5 and other inflammatory factors to activate macrophages and eliminate viruses. 7.CD4^+^ T cells stimulate B cells to produce neutralizing antibodies, thus neutralizing invading pathogens.

An mRNA vaccine injected into the body is ingested by antigen-presenting cells (APCs), where the antigen-encoding mRNA is released from its carrier into the cytoplasm. Following recognition of the mRNA by the ribosome it is translated into antigenic protein, just as occurs with endogenous mRNA. The antigenic proteins are then broken down into small antigenic fragments by the proteasome complex. The decomposed antigen fragments are captured by MHC-I molecules and presented to CD8^+^ T cells, which in response release granzyme and perforin to lyse the infected cells. Antigen fragments decomposed by the proteasome complex are also recognized by MHC-II molecules and presented to CD4^+^ T cells. Th1 cells secrete IFN-γ and tumor necrosis factor-α, and Th2 cells secrete interleukin (IL)-4, IL-5, and other inflammatory factors to activate macrophages and eliminate viruses. CD4^+^ T cells can also stimulate B cells to produce neutralizing antibodies that neutralize invading pathogens (Figure 3).

**Figure 4:**
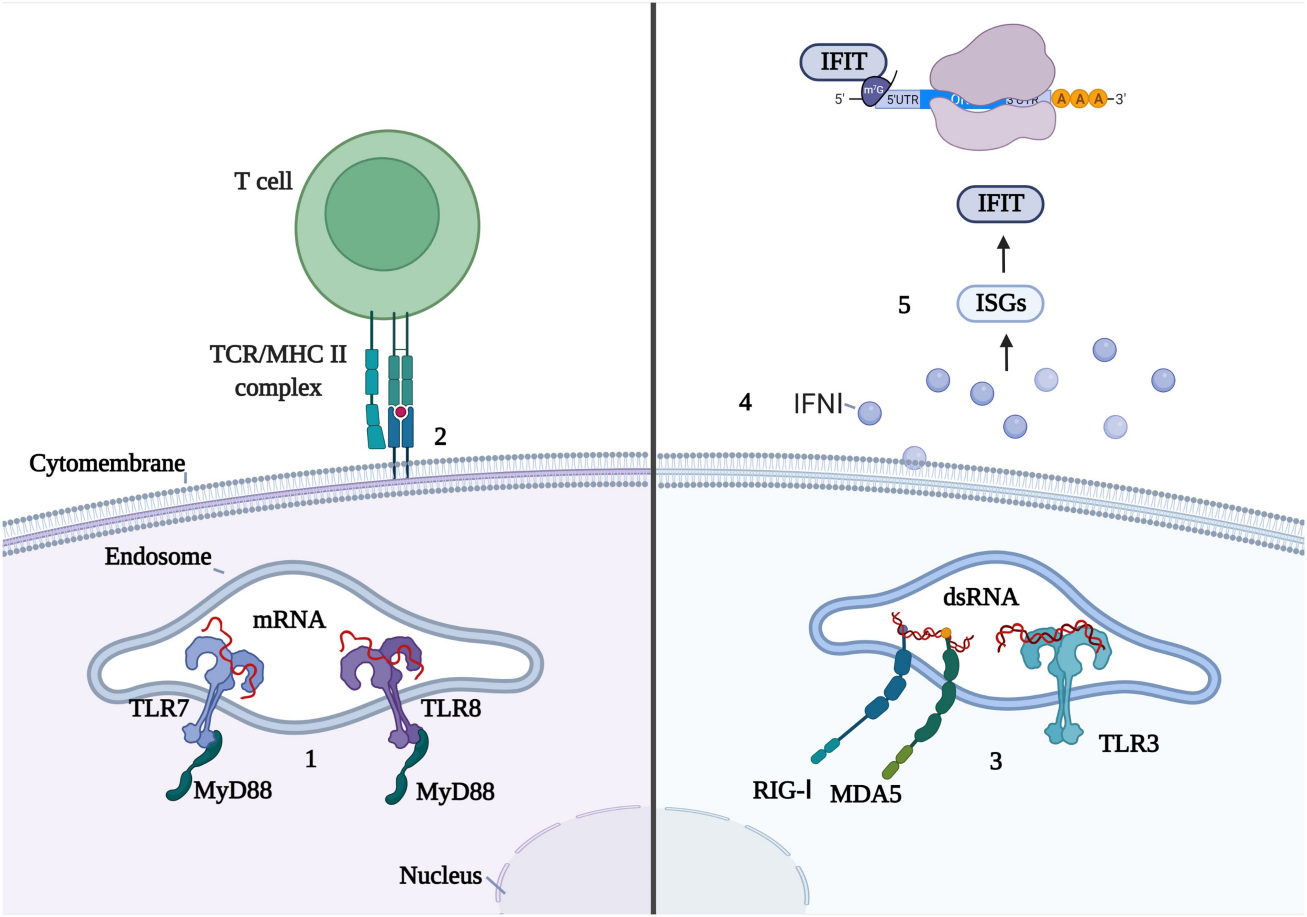
Double-edged sword of innate immune response with mRNA vaccine. 1.TLR7 and TLR8 recognize single chain mRNA and activate MyD88. 2. Innate immune cells activate CD4 + T cells through MHC II molecules to activate adaptive immune responses. 3.RIG-I, MDA5, and TLR3 recognize dsRNA. 4. Innate immune cells release IFNI and activate ISG. 5. ISG overexpresses IFIT, which binds to the mRNA cap or elF3 and prevents the mRNA translation process.

However, the entry of exogenous mRNA and its by-products into the body can trigger innate immunity, which not only affects translation efficiency but may also influence the efficacy and safety of the vaccine (Figure 4). As noted above, IVT mRNAs and their by-products are recognized by PRRs, mainly RIGI-like receptors (RLRs) and endosomal Toll-like receptors. TLR-7 and TLR-8 preferentially bind G-rich single-stranded RNA (Heil et al., 2004). Both receptors are part of the MyD88 (myeloid differentiation marker 88) pathway (Linares-Fernández et al., 2020a) and bind to uridine in a uracil-and ribose-dependent manner. Because the same binding sites on uracil are present on chemically modified uridine, such as pseudouracil (ψ), the use of modified bases, such as pseuduracil, can activate the innate immune response and in turn, the adaptive immune response. dsRNAs are generated during IVT and they activate TLR-3, RIG-I, and MDA5, leading to the production of type I IFN (Patel and García-Sastre, 2014), thereby inducing the expression of IFN-stimulating genes (ISGs; Linares-Fernández et al., 2020a) and the high-level expression of IFIT (IFN-inducible protein with tetratricoid repeats). IFIT bind to the cap end of RNA (Kumar et al., 2014) or interact with the eukaryotic cell initiation factor eIF3 to block mRNA translation.

### Autoimmune diseases

In September 2021, Sozen et al. reported five cases of fever, neck pain, weakness, and tremors in individuals who, a few days before, had received the BNT162b2 mRNA vaccine and were not infected with SARS-CoV-2. Together, the clinical, imaging, and laboratory findings led to a diagnosis of subacute thyroiditis, considered an autoimmune disease caused by the vaccine.

In December 2021, Ibrahim et al. (2022) reported a case of eosinophilic granuloma complicated with poly vasculitis following vaccination with Moderna’s mRNA-1273. The patient presented with progressive weakness and paresthesia in the upper and lower limbs, peripheral eosinophilia, and elevated anti-myeloperoxidase antibodies. Nerve and muscle biopsies showed focal vasculitis with eosinophilic infiltration. Symptoms improved significantly with steroid and nonsteroidal medications.

In January 2022, Boettler et al. (2022) reported a case of acute hepatitis in a patient who had been vaccinated 2–3 weeks earlier with BNT162b2. Acute mixed hepatocellular hepatitis occurred after the first inoculation, and severe hepatitis after the second. The diagnosis was autoimmune hepatitis. Improvement after treatment was followed by a relapse. A comprehensive immunological evaluation of the inflammatory infiltrates in the liver revealed the presence of highly activated cytotoxic CD8 T cells, including a SARS-CoV-2-specific CD8 T-cell population also detected in the periphery. These results indicate that post-vaccination hepatitis involves vaccine-induced antigen-specific immune responses and differs from true autoimmune hepatitis in its histological characteristics. Thus, hepatitis after one dose of BNT162b2 vaccine may be triggered by a history of liver autoimmunity.

In addition to these sporadic cases, Guillain–Barre syndrome (GBS) has been reported following vaccination with ChAdOx1 NCOV-19 (Oxford/AstraZeneca), AD26.coV 2.s (Johnson & Johnson’s Janssen), BNT162b2 (Pfizer-BioNTech), and mRNA-1273 (Moderna). GBS is an acute, immune-mediated heterogeneous disorder of the nervous system that affects the peripheral nerves and nerve roots. Cases of GBS have been reported following surgery, trauma, and vaccination. Some studies have found a slightly higher incidence of GBS after influenza vaccination, but epidemiological studies have not found a direct link between the vaccine and GBS (Nagalli and Shankar Kikkeri, 2022). Similarly, the cases of GBS after the mRNA vaccine were sporadic, and a specific association with the vaccine has not been determined.

While these adverse reactions are mostly autoimmune diseases, there is no direct evidence that they are related to the COVID-19 mRNA vaccine. However, further studies are needed and clinicians must be sensitive to these cases, as autoimmune diseases require early detection and treatment to prevent long-term complications.

## Future opportunities for mRNA vaccines in infectious diseases

As mRNA vaccine technology has gradually matured, its advantages in the field of infectious disease prevention have become increasingly obvious. Traditional inactivated vaccines cannot be produced fast enough to meet sudden outbreaks of infections such as COVID-19, and the conditions to ensure their production safety result in further delays. The development of live attenuated vaccines is also time-consuming and their isolation, culture, and production are high-risk. The main disadvantages of recombinant protein vaccines are low antigen expression and a complex production process. Moreover, all three of these traditional vaccines have only weak immunogenicity such that they require an adjuvant and multiple vaccinations to enhance immunity. By contrast, mRNA vaccines are produced *in vitro*, by transcribing the sequence encoding the target antigen. They are also independent of cell amplification, resulting in shorter manufacturing times, a safer preparation process, and the possibility of rapid mass production in response to public health emergencies, such as the COVID-19 pandemic. In addition to the diseases discussed herein, mRNA vaccines are being studied for their possible use against infection with *Plasmodium*, Ebola virus, and other diseases. Some of these vaccine candidates are expected to soon enter clinical trials.

Current data show that mRNA vaccines are relatively safe. The number of adverse events in clinical trials and in the vaccinated public has thus far been very small, but the long-term effects are still unknown. The main challenges in the use of mRNA vaccines in infectious diseases are the need for more stable mRNA delivery systems, a weakened antigenic immune response over time, and as noted above, the development of post-vaccination autoimmune diseases. In conclusion, mRNA vaccines hold great promise as a novel weapon in the fight against infectious diseases.

## Author contributions

YT and ZD wrote the manuscript and drafted the figures. PY proofreads the manuscript and revises it. All authors contributed to the article and approved the submitted version.

## Conflict of interest

The authors declare that the research was conducted in the absence of any commercial or financial relationships that could be construed as a potential conflict of interest.

## Publisher’s note

All claims expressed in this article are solely those of the authors and do not necessarily represent those of their affiliated organizations, or those of the publisher, the editors and the reviewers. Any product that may be evaluated in this article, or claim that may be made by its manufacturer, is not guaranteed or endorsed by the publisher.

## References

[ref1] Aldrich C. Leroux-Roels I. Huang K. B. Bica M. A. Loeliger E. Schoenborn-Kellenberger O.. (2021). Proof-of-concept of a low-dose unmodified mRNA-based rabies vaccine formulated with lipid nanoparticles in human volunteers: a phase 1 trial. Vaccine 39, 1310–1318. doi: 10.1016/j.vaccine.2020.12.070, PMID: 33487468 PMC7825876

[ref2] Al-Saif M. Khabar K. S. (2012). UU/UA dinucleotide frequency reduction in coding regions results in increased mRNA stability and protein expression. Mol. Ther. 20, 954–959. doi: 10.1038/mt.2012.29, PMID: 22434136 PMC3345983

[ref3] Anderson B. R. Muramatsu H. Jha B. K. Silverman R. H. Weissman D. Karikó K. (2011). Nucleoside modifications in RNA limit activation of 2′-5′-oligoadenylate synthetase and increase resistance to cleavage by RNase L. Nucleic Acids Res. 39, 9329–9338. doi: 10.1093/nar/gkr586, PMID: 21813458 PMC3241635

[ref4] Anderson E. J. Rouphael N. G. Widge A. T. Jackson L. A. Roberts P. C. Makhene M.. (2020). Safety and immunogenicity of SARS-CoV-2 mRNA-1273 vaccine in older adults. N. Engl. J. Med. 383, 2427–2438. doi: 10.1056/NEJMoa2028436, PMID: 32991794 PMC7556339

[ref5] Asrani K. H. Cheng L. Cheng C. J. Subramanian R. R. (2018). Arginase I mRNA therapy - a novel approach to rescue arginase 1 enzyme deficiency. RNA Biol. 15, 914–922. doi: 10.1080/15476286.2018.1475178, PMID: 29923457 PMC6161738

[ref6] Baden L. R. El Sahly H. M. Essink B. Kotloff K. Frey S. Novak R.. (2021). Efficacy and safety of the mRNA-1273 SARS-CoV-2 vaccine. N. Engl. J. Med. 384, 403–416. doi: 10.1056/NEJMoa2035389, PMID: 33378609 PMC7787219

[ref7] Baiersdörfer M. Boros G. Muramatsu H. Mahiny A. Vlatkovic I. Sahin U.. (2019a). A facile method for the removal of ds RNA contaminant from in vitro-transcribed mRNA. Mol. Ther. Nucleic Acids 15, 26–35. doi: 10.1016/j.omtn.2019.02.018, PMID: 30933724 PMC6444222

[ref8] Baiersdörfer M. Boros G. Muramatsu H. Mahiny A. Vlatkovic I. Sahin U.. (2019b). A facile method for the removal of dsRNA contaminant from *in vitro*-transcribed mRNA. Mol. Ther. Nucleic Acids 15, 26–35. doi: 10.1016/j.omtn.2019.02.018, PMID: 30933724 PMC6444222

[ref9] Barré-Sinoussi F. Chermann J. C. Rey F. Nugeyre M. T. Chamaret S. Gruest J.. (1983). Isolation of a T-lymphotropic retrovirus from a patient at risk for acquired immune deficiency syndrome (AIDS). Science 220, 868–871. doi: 10.1126/science.6189183 6189183

[ref10] Beck J. D. Reidenbach D. Salomon N. Sahin U. Türeci Ö. Vormehr M.. (2021). mRNA therapeutics in cancer immunotherapy. Mol. Cancer 20:69. doi: 10.1186/s12943-021-01348-0, PMID: 33858437 PMC8047518

[ref11] Blount R. E. Jr. Morris J. A. Savage R. E. (1956). Recovery of cytopathogenic agent from chimpanzees with coryza. Proc. Soc. Exp. Biol. Med. 92, 544–549. doi: 10.3181/00379727-92-22538, PMID: 13359460

[ref12] Boettler T. Csernalabics B. Salié H. Luxenburger H. Wischer L. Alizei E. S.. (2022). SARS-CoV-2 vaccination can elicit a CD8 T-cell dominant hepatitis. J. Hepatol. 77, 653–659. doi: 10.1016/j.jhep.2022.03.040, PMID: 35461912 PMC9021033

[ref13] Bogers W. M. Oostermeijer H. Mooij P. Koopman G. Verschoor E. J. Davis D.. (2015). Potent immune responses in rhesus macaques induced by nonviral delivery of a self-amplifying RNA vaccine expressing HIV type 1 envelope with a cationic nanoemulsion. J. Infect. Dis. 211, 947–955. doi: 10.1093/infdis/jiu522, PMID: 25234719 PMC4416123

[ref14] Brasil P. Pereira J. P. Jr. Moreira M. E. Ribeiro Nogueira R. M. Damasceno L. Wakimoto M.. (2016). Zika virus infection in pregnant women in Rio de Janeiro. N. Engl. J. Med. 375, 2321–2334. doi: 10.1056/NEJMoa1602412, PMID: 26943629 PMC5323261

[ref15] Buchbinder S. P. Mehrotra D. V. Duerr A. Fitzgerald D. W. Mogg R. Li D.. (2008). Efficacy assessment of a cell-mediated immunity HIV-1 vaccine (the step study): a double-blind, randomised, placebo-controlled, test-of-concept trial. Lancet 372, 1881–1893. doi: 10.1016/s0140-6736(08)61591-3, PMID: 19012954 PMC2721012

[ref16] Cai X. Li J. J. Liu T. Brian O. Li J. (2021). Infectious disease mRNA vaccines and a review on epitope prediction for vaccine design. Brief. Funct. Genomics 20, 289–303. doi: 10.1093/bfgp/elab027, PMID: 34089044 PMC8194884

[ref17] Carralot J. P. Probst J. Hoerr I. Scheel B. Teufel R. Jung G.. (2004). Polarization of immunity induced by direct injection of naked sequence-stabilized mRNA vaccines. Cell. Mol. Life Sci. 61, 2418–2424. doi: 10.1007/s00018-004-4255-0, PMID: 15378210 PMC7079797

[ref18] CDC Seasonal Flu Vaccine Effectiveness Studies (2021-2022). U.S. flu season: Preliminary in-season burden estimates [online]. Available at: https://www.cdc.gov/flu/about/burden/preliminary-in-season-estimates.htm?msclkid=ffacd4dbce3c11eca39a9738d63f1f64 (Accessed June 17,2022).

[ref19] Chakraborty C. Sharma A. R. Bhattacharya M. Lee S. S. (2021). From COVID-19 to cancer mRNA vaccines: moving from bench to Clinic in the Vaccine Landscape. Front. Immunol. 12:679344. doi: 10.3389/fimmu.2021.679344, PMID: 34305909 PMC8293291

[ref20] Chen G.-L. Li X.-F. Dai X.-H. Li N. Cheng M.-L. Huang Z.. (2022). Safety and immunogenicity of the SARS-CoV-2 ARCoV mRNA vaccine in Chinese adults: a randomised, double-blind, placebo-controlled, phase 1 trial. Lancet. Microbe 3, e193–e202. doi: 10.1016/S2666-5247(21)00280-9, PMID: 35098177 PMC8786321

[ref21] Chivukula S. Plitnik T. Tibbitts T. Karve S. Dias A. Zhang D.. (2021). Development of multivalent mRNA vaccine candidates for seasonal or pandemic influenza. NPJ Vaccines 6:153. doi: 10.1038/s41541-021-00420-6, PMID: 34916519 PMC8677760

[ref22] Corbett K. S. Edwards D. K. Leist S. R. Abiona O. M. Boyoglu-Barnum S. Gillespie R. A.. (2020). SARS-CoV-2 mRNA vaccine design enabled by prototype pathogen preparedness. Nature 586, 567–571. doi: 10.1038/s41586-020-2622-0, PMID: 32756549 PMC7581537

[ref23] de Jong W. Aerts J. Allard S. Brander C. Buyze J. Florence E.. (2019). iHIVARNA phase IIa, a randomized, placebo-controlled, double-blinded trial to evaluate the safety and immunogenicity of iHIVARNA-01 in chronically HIV-infected patients under stable combined antiretroviral therapy. Trials 20:361. doi: 10.1186/s13063-019-3409-1, PMID: 31208472 PMC6580477

[ref24] Delgado M. F. Coviello S. Monsalvo A. C. Melendi G. A. Hernandez J. Z. Batalle J. P.. (2009). Lack of antibody affinity maturation due to poor toll-like receptor stimulation leads to enhanced respiratory syncytial virus disease. Nat. Med. 15, 34–41. doi: 10.1038/nm.1894, PMID: 19079256 PMC2987729

[ref25] Febbraio M. A. Reibe S. Shalapour S. Ooi G. J. Watt M. J. Karin M. (2019). Preclinical models for studying NASH-driven HCC: how useful are they? Cell Metab. 29, 18–26. doi: 10.1016/j.cmet.2018.10.012, PMID: 30449681 PMC6326872

[ref26] Foulon I. Naessens A. Foulon W. Casteels A. Gordts F. (2008). A 10-year prospective study of sensorineural hearing loss in children with congenital cytomegalovirus infection. J. Pediatr. 153, 84–88. doi: 10.1016/j.jpeds.2007.12.049, PMID: 18571542

[ref27] Freyn A. W. Ramos da Silva J. Rosado V. C. Bliss C. M. Pine M. Mui B. L.. (2020). A multi-targeting, nucleoside-modified mRNA influenza virus vaccine provides broad protection in mice. Mol. Ther. 28, 1569–1584. doi: 10.1016/j.ymthe.2020.04.018, PMID: 32359470 PMC7335735

[ref28] Friedman-Kien A. E. (1981). Disseminated Kaposi's sarcoma syndrome in young homosexual men. J. Am. Acad. Dermatol. 5, 468–471. doi: 10.1016/s0190-9622(81)80010-2, PMID: 7287964

[ref29] Fu X. Chen T. Song Y. Feng C. Chen H. Zhang Q.. (2021). mRNA delivery by a pH-responsive DNA Nano-hydrogel. Small 17:e2101224. doi: 10.1002/smll.202101224, PMID: 34145748

[ref30] Gómez C. E. Perdiguero B. Usero L. Marcos-Villar L. Miralles L. Leal L.. (2021). Enhancement of the HIV-1-specific immune response induced by an mRNA vaccine through boosting with a poxvirus MVA vector expressing the same antigen. Vaccine 9. doi: 10.3390/vaccines9090959, PMID: 34579196 PMC8473054

[ref31] Haabeth O. A. W. Lohmeyer J. J. K. Sallets A. Blake T. R. Sagiv-Barfi I. Czerwinski D. K.. (2021). An mRNA SARS-CoV-2 vaccine employing charge-altering releasable transporters with a TLR-9 agonist induces neutralizing antibodies and T cell memory. ACS Cent. Sci. 7, 1191–1204. doi: 10.1021/acscentsci.1c00361, PMID: 34341771 PMC8265720

[ref32] Heil F. Hemmi H. Hochrein H. Ampenberger F. Kirschning C. Akira S.. (2004). Species-specific recognition of single-stranded RNA via toll-like receptor 7 and 8. Science 303, 1526–1529. doi: 10.1126/science.1093620, PMID: 14976262

[ref33] Hekele A. Bertholet S. Archer J. Gibson D. G. Palladino G. Brito L. A.. (2013). Rapidly produced SAM(^®^) vaccine against H7N9 influenza is immunogenic in mice. Emerg. Microbes Infect. 2:e52. doi: 10.1038/emi.2013.54, PMID: 26038486 PMC3821287

[ref34] Hemachudha T. Ugolini G. Wacharapluesadee S. Sungkarat W. Shuangshoti S. Laothamatas J. (2013). Human rabies: neuropathogenesis, diagnosis, and management. Lancet Neurol. 12, 498–513. doi: 10.1016/s1474-4422(13)70038-3, PMID: 23602163

[ref35] Ibrahim H. Alkhatib A. Meysami A. (2022). Eosinophilic granulomatosis with polyangiitis diagnosed in an elderly female after the second dose of mRNA vaccine against COVID-19. Cureus 14:e21176. doi: 10.7759/cureus.21176, PMID: 35165624 PMC8831231

[ref36] Izikson R. Brune D. Bolduc J.-S. Bourron P. Fournier M. Moore T. M.. (2022). Safety and immunogenicity of a high-dose quadrivalent influenza vaccine administered concomitantly with a third dose of the mRNA-1273 SARS-CoV-2 vaccine in adults aged ≥65 years: a phase 2, randomised, open-label study. Lancet Respir. Med. 10, 392–402. doi: 10.1016/S2213-2600(21)00557-9, PMID: 35114141 PMC8803382

[ref37] Jackson L. A. Anderson E. J. Rouphael N. G. Roberts P. C. Makhene M. Coler R. N.. (2020). An mRNA vaccine against SARS-CoV-2- preliminary report. N. Engl. J. Med. 383, 1920–1931. doi: 10.1056/NEJMoa2022483, PMID: 32663912 PMC7377258

[ref38] Jemielity J. Fowler T. Zuberek J. Stepinski J. Lewdorowicz M. Niedzwiecka A.. (2003). Novel "anti-reverse" cap analogs with superior translational properties. RNA 9, 1108–1122. doi: 10.1261/rna.5430403, PMID: 12923259 PMC1370475

[ref39] Julie (2021). Moderna developing mRNA-1073 COVID booster, flu combo vaccine [online]. Available at: https://www.al.com/news/2021/09/moderna-developing-covid-19-booster-flu-combo-vaccine.html (Accessed June 17,2022).

[ref40] Kallen K. J. Heidenreich R. Schnee M. Petsch B. Schlake T. Thess A.. (2013a). A novel, disruptive vaccination technology: self-adjuvanted RNActive(^®^) vaccines. Hum. Vaccin. Immunother. 9, 2263–2276. doi: 10.4161/hv.25181, PMID: 23921513 PMC3906413

[ref41] Karikó K. Buckstein M. Ni H. Weissman D. (2005). Suppression of RNA recognition by toll-like receptors: the impact of nucleoside modification and the evolutionary origin of RNA. Immunity 23, 165–175. doi: 10.1016/j.immuni.2005.06.008, PMID: 16111635

[ref42] Kim H. W. Canchola J. G. Brandt C. D. Pyles G. Chanock R. M. Jensen K.. (1969). Respiratory syncytial virus disease in infants despite prior administration of antigenic inactivated vaccine. Am. J. Epidemiol. 89, 422–434. doi: 10.1093/oxfordjournals.aje.a120955, PMID: 4305198

[ref43] Kimchi-Sarfaty C. Oh J. M. Kim I. W. Sauna Z. E. Calcagno A. M. Ambudkar S. V.. (2007). A "silent" polymorphism in the MDR1 gene changes substrate specificity. Science 315, 525–528. doi: 10.1126/science.1135308, PMID: 17185560

[ref44] Kreiter S. Selmi A. Diken M. Koslowski M. Britten C. M. Huber C.. (2010a). Intranodal vaccination with naked antigen-encoding RNA elicits potent prophylactic and therapeutic antitumoral immunity. Cancer Res. 70, 9031–9040. doi: 10.1158/0008-5472.Can-10-0699, PMID: 21045153

[ref45] Kumar P. Sweeney T. R. Skabkin M. A. Skabkina O. V. Hellen C. U. Pestova T. V. (2014). Inhibition of translation by IFIT family members is determined by their ability to interact selectively with the 5′-terminal regions of cap0-, cap1-and 5'ppp-mRNAs. Nucleic Acids Res. 42, 3228–3245. doi: 10.1093/nar/gkt1321, PMID: 24371270 PMC3950709

[ref46] Kwon H. Kim M. Seo Y. Moon Y. S. Lee H. J. Lee K.. (2018a). Emergence of synthetic mRNA: in vitro synthesis of mRNA and its applications in regenerative medicine. Biomaterials 156, 172–193. doi: 10.1016/j.biomaterials.2017.11.034, PMID: 29197748

[ref47] Li B. Zeng C. Dong Y. (2018). Design and assessment of engineered CRISPR-Cpf1 and its use for genome editing. Nat. Protoc. 13, 899–914. doi: 10.1038/nprot.2018.004, PMID: 29622802 PMC6383568

[ref48] Linares-Fernández S. Lacroix C. Exposito J. Y. Verrier B. (2020a). Tailoring mRNA vaccine to balance innate/adaptive immune response. Trends Mol. Med. 26, 311–323. doi: 10.1016/j.molmed.2019.10.002, PMID: 31699497

[ref49] Liu G. Zhu M. Zhao X. Nie G. (2021). Nanotechnology-empowered vaccine delivery for enhancing CD8(+) T cells-mediated cellular immunity. Adv. Drug Deliv. Rev. 176:113889. doi: 10.1016/j.addr.2021.113889, PMID: 34364931

[ref50] Masison D. C. Blanc A. Ribas J. C. Carroll K. Sonenberg N. Wickner R. B. (1995). Decoying the cap-mRNA degradation system by a double-stranded RNA virus and poly(A)-mRNA surveillance by a yeast antiviral system. Mol. Cell. Biol. 15, 2763–2771. doi: 10.1128/mcb.15.5.2763, PMID: 7739557 PMC230507

[ref51] Mbatha L. S. Maiyo F. Daniels A. Singh M. (2021a). Dendrimer-coated gold nanoparticles for efficient Folate-targeted mRNA delivery *in vitro*. Pharmaceutics 13. doi: 10.3390/pharmaceutics13060900, PMID: 34204271 PMC8235267

[ref52] Miao L. Zhang Y. Huang L. (2021). mRNA vaccine for cancer immunotherapy. Mol. Cancer 20:41. doi: 10.1186/s12943-021-01335-5, PMID: 33632261 PMC7905014

[ref53] Mu X. Greenwald E. Ahmad S. Hur S. (2018). An origin of the immunogenicity of *in vitro* transcribed RNA. Nucleic Acids Res. 46, 5239–5249. doi: 10.1093/nar/gky177, PMID: 29534222 PMC6007322

[ref54] Mu X. Hur S. (2021). Immunogenicity of in vitro-transcribed RNA. Acc. Chem. Res. 54, 4012–4023. doi: 10.1021/acs.accounts.1c00521, PMID: 34677064 PMC9127547

[ref55] Mulligan M. J. Lyke K. E. Kitchin N. Absalon J. Gurtman A. Lockhart S.. (2020). Phase I/II study of COVID-19 RNA vaccine BNT162b1 in adults. Nature 586, 589–593. doi: 10.1038/s41586-020-2639-4, PMID: 32785213

[ref56] Nagalli S. Shankar Kikkeri N. (2022). Sub-acute onset of Guillain-Barré syndrome post-mRNA-1273 vaccination: a case report. SN Compr. Clin. Med. 4:41. doi: 10.1007/s42399-022-01124-1, PMID: 35071987 PMC8764171

[ref57] Nam H. H. Ison M. G. (2019). Respiratory syncytial virus infection in adults. BMJ 366:l5021. doi: 10.1136/bmj.l5021 31506273

[ref58] Ngwuta J. O. Chen M. Modjarrad K. Joyce M. G. Kanekiyo M. Kumar A.. (2015). Prefusion F-specific antibodies determine the magnitude of RSV neutralizing activity in human sera. Sci. Transl. Med. 7:309ra162. doi: 10.1126/scitranslmed.aac4241, PMID: 26468324 PMC4672383

[ref59] Pardi N. Muramatsu H. Weissman D. Karikó K. (2013). In vitro transcription of long RNA containing modified nucleosides. Methods Mol. Biol. 969, 29–42. doi: 10.1007/978-1-62703-260-5_2, PMID: 23296925

[ref60] Pasquinelli A. E. Dahlberg J. E. Lund E. (1995). Reverse 5′ caps in RNAs made *in vitro* by phage RNA polymerases. RNA 1, 957–967. PMID: 8548660 PMC1369344

[ref61] Passmore L. A. Coller J. (2022). Roles of mRNA poly(A) tails in regulation of eukaryotic gene expression. Nat. Rev. Mol. Cell Biol. 23, 93–106. doi: 10.1038/s41580-021-00417-y, PMID: 34594027 PMC7614307

[ref62] Patel J. R. García-Sastre A. (2014). Activation and regulation of pathogen sensor RIG-I. Cytokine Growth Factor Rev. 25, 513–523. doi: 10.1016/j.cytogfr.2014.08.005, PMID: 25212896

[ref63] Payne R. P. Longet S. Austin J. A. Skelly D. T. Dejnirattisai W. Adele S.. (2021). Immunogenicity of standard and extended dosing intervals of BNT162b2 mRNA vaccine. Cells 184, 5699–5714.e11. doi: 10.1016/j.cell.2021.10.011, PMID: 34735795 PMC8519781

[ref64] Petsch B. Schnee M. Vogel A. B. Lange E. Hoffmann B. Voss D.. (2012). Protective efficacy of in vitro synthesized, specific mRNA vaccines against influenza A virus infection. Nat. Biotechnol. 30, 1210–1216. doi: 10.1038/nbt.2436, PMID: 23159882

[ref65] Ramanan P. Razonable R. R. (2013). Cytomegalovirus infections in solid organ transplantation: a review. Infect. Chemother. 45, 260–271. doi: 10.3947/ic.2013.45.3.260, PMID: 24396627 PMC3848521

[ref66] Ramanathan A. Robb G. B. Chan S. H. (2016). mRNA capping: biological functions and applications. Nucleic Acids Res. 44, 7511–7526. doi: 10.1093/nar/gkw551, PMID: 27317694 PMC5027499

[ref67] Ren J. Cao Y. Li L. Wang X. Lu H. Yang J.. (2021a). Self-assembled polymeric micelle as a novel mRNA delivery carrier. J. Control. Release 338, 537–547. doi: 10.1016/j.jconrel.2021.08.061, PMID: 34481924 PMC8411660

[ref68] Sahin U. Karikó K. Türeci Ö. (2014). mRNA-based therapeutics--developing a new class of drugs. Nat. Rev. Drug Discov. 13, 759–780. doi: 10.1038/nrd4278, PMID: 25233993

[ref69] Sahin U. Muik A. Derhovanessian E. Vogler I. Kranz L. M. Vormehr M.. (2020). COVID-19 vaccine BNT162b1 elicits human antibody and T1 T cell responses. Nature 586, 594–599. doi: 10.1038/s41586-020-2814-7, PMID: 32998157

[ref70] Schmidt M. R. McGinnes L. W. Kenward S. A. Willems K. N. Woodland R. T. Morrison T. G. (2012). Long-term and memory immune responses in mice against Newcastle disease virus-like particles containing respiratory syncytial virus glycoprotein ectodomains. J. Virol. 86, 11654–11662. doi: 10.1128/jvi.01510-12, PMID: 22896618 PMC3486317

[ref71] Schnee M. Vogel A. B. Voss D. Petsch B. Baumhof P. Kramps T.. (2016). An mRNA vaccine encoding rabies virus glycoprotein induces protection against lethal infection in mice and correlates of protection in adult and newborn pigs. PLoS Negl. Trop. Dis. 10:e0004746. doi: 10.1371/journal.pntd.0004746, PMID: 27336830 PMC4918980

[ref72] Skowronski D. M. De Serres G. (2021a). Safety and efficacy of the BNT162b2 mRNA Covid-19 vaccine. N. Engl. J. Med. 384, 1576–1578. doi: 10.1056/NEJMc2036242 33596348

[ref73] Staff (2022). mRNA-1345 respiratory syncytial virus (RSV) vaccine description for 2022 [online]. Available at: https://www.precisionvaccinations.com/vaccines/mrna-1345-respiratory-syncytial-virus-rsv-vaccine (Accessed June 17,2022).

[ref74] Standaert B. Rappuoli R. (2017). Towards a more comprehensive approach for a total economic assessment of vaccines?: 1. The building blocks for a health economic assessment of vaccination. J. Mark Access. Health Policy 5:1335162. doi: 10.1080/20016689.2017.1335162, PMID: 29785251 PMC5956291

[ref75] Stepinski J. Waddell C. Stolarski R. Darzynkiewicz E. Rhoads R. E. (2001). Synthesis and properties of mRNAs containing the novel "anti-reverse" cap analogs 7-methyl(3'-O-methyl)GpppG and 7-methyl (3'-deoxy)GpppG. RNA 7, 1486–1495. doi: 10.1017/S1355838201014078 11680853 PMC1370192

[ref76] Stokes A. Pion J. Binazon O. Laffont B. Bigras M. Dubois G.. (2020). Nonclinical safety assessment of repeated administration and biodistribution of a novel rabies self-amplifying mRNA vaccine in rats. Regul. Toxicol. Pharmacol. 113:104648. doi: 10.1016/j.yrtph.2020.104648, PMID: 32240713

[ref77] Sweeney R. Fan Q. Yao M. C. (1996). Antisense ribosomes: rRNA as a vehicle for antisense RNAs. Proc. Natl. Acad. Sci. U. S. A. 93, 8518–8523. doi: 10.1073/pnas.93.16.8518, PMID: 8710902 PMC38704

[ref78] Teo S. P. (2021). Review of COVID-19 mRNA vaccines: BNT162b2 and mRNA-1273. J. Pharm. Pract.:8971900211009650. doi: 10.1177/08971900211009650 33840294

[ref79] Tusup M. Läuchli S. Jarzebska N. T. French L. E. Chang Y. T. Vonow-Eisenring M.. (2021). mRNA-based anti-TCR CDR3 tumour vaccine for T-cell lymphoma. Pharmaceutics 13. doi: 10.3390/pharmaceutics13071040, PMID: 34371731 PMC8308944

[ref80] Valentin A. Bergamaschi C. Rosati M. Angel M. Burns R. Agarwal M.. (2022). Comparative immunogenicity of an mRNA/LNP and a DNA vaccine targeting HIV conserved elements in macaques. Front. Immunol. 13:945706. doi: 10.3389/fimmu.2022.945706, PMID: 35935984 PMC9355630

[ref81] Vogel A. B. Kanevsky I. Che Y. Swanson K. A. Muik A. Vormehr M.. (2020). “BNT162b vaccines are immunogenic and protect non-human primates against SARS-CoV-2.” *bioRxiv* doi: 10.1101/2020.12.11.421008 [Epub ahead of preprint].

[ref82] Wadhwa A. Aljabbari A. Lokras A. Foged C. Thakur A. (2020). Opportunities and challenges in the delivery of mRNA-based vaccines. Pharmaceutics 12. doi: 10.3390/pharmaceutics12020102, PMID: 32013049 PMC7076378

[ref83] Watson J. M. Pebody R. G. (2011). Pandemic influenza vaccines. BMJ (Clinical Research ed.) 342:d545. doi: 10.1136/bmj.d545 21303882

[ref84] Weissman D. Pardi N. Muramatsu H. Karikó K. (2013). HPLC purification of *in vitro* transcribed long RNA. Methods Mol. Biol. 969, 43–54. doi: 10.1007/978-1-62703-260-5_3, PMID: 23296926

[ref85] WHO (2022). World Health Statistics 2022: Monitoring Health for the SDGs, Sustainable Development Goals. Geneva: WHO.

[ref86] Wolff J. A. Malone R. W. Williams P. Chong W. Acsadi G. Jani A.. (1990). Direct gene transfer into mouse muscle in vivo. Science 247, 1465–1468. doi: 10.1126/science.1690918 1690918

[ref87] Wrapp D. Wang N. Corbett K. S. Goldsmith J. A. Hsieh C.-L. Abiona O.. (2020). Cryo-EM Structure of the 2019-nCoV Spike in the prefusion conformation. Science 367, 1260–1263. doi: 10.1126/science.abb2507, PMID: 32075877 PMC7164637

[ref88] Xu S. Yang K. Li R. Zhang L. (2020). mRNA vaccine era-mechanisms, drug platform and clinical prospection. Int. J. Mol. Sci. 21. doi: 10.3390/ijms21186582, PMID: 32916818 PMC7554980

[ref89] Yang R. Deng Y. Huang B. Huang L. Lin A. Li Y.. (2021). A core-shell structured COVID-19 mRNA vaccine with favorable biodistribution pattern and promising immunity. Signal Transduct. Target. Ther. 6:213. doi: 10.1038/s41392-021-00634-z, PMID: 34059617 PMC8165147

[ref90] Zhang L. Jackson C. B. Mou H. Ojha A. Peng H. Quinlan B. D.. (2020a). SARS-CoV-2 spike-protein D614G mutation increases virion spike density and infectivity. Nat. Commun. 11:6013. doi: 10.1038/s41467-020-19808-4, PMID: 33243994 PMC7693302

[ref91] Zhang P. Narayanan E. Liu Q. Tsybovsky Y. Boswell K. Ding S.. (2021a). A multiclade env-gag VLP mRNA vaccine elicits tier-2 HIV-1-neutralizing antibodies and reduces the risk of heterologous SHIV infection in macaques. Nat. Med. 27, 2234–2245. doi: 10.1038/s41591-021-01574-5, PMID: 34887575

[ref92] Zhang M. Sun J. Li M. Jin X. (2020b). Modified mRNA-LNP vaccines confer protection against experimental DENV-2 infection in mice. Mol. Ther. Methods Clin. Dev. 18, 702–712. doi: 10.1016/j.omtm.2020.07.013, PMID: 32913878 PMC7452130

[ref93] Zhao M. Li M. Zhang Z. Gong T. Sun X. (2016). Induction of HIV-1 gag specific immune responses by cationic micelles mediated delivery of gag mRNA. Drug Deliv. 23, 2596–2607. doi: 10.3109/10717544.2015.1038856, PMID: 26024387

[ref94] Zhao X. Pan X. Wang Y. Zhang Y. (2021). Targeting neoantigens for cancer immunotherapy. Biomark Res. 9:61. doi: 10.1186/s40364-021-00315-7, PMID: 34321091 PMC8317330

[ref95] Zhong F. Cao W. Chan E. Tay P. N. Cahya F. F. Zhang H.. (2005). Deviation from major codons in the toll-like receptor genes is associated with low toll-like receptor expression. Immunology 114, 83–93. doi: 10.1111/j.1365-2567.2004.02007.x, PMID: 15606798 PMC1782050

[ref96] Zhuang X. Qi Y. Wang M. Yu N. Nan F. Zhang H.. (2020). mRNA vaccines encoding the HA protein of influenza A H1N1 virus delivered by cationic lipid nanoparticles induce protective immune responses in mice. Vaccine 8. doi: 10.3390/vaccines8010123, PMID: 32164372 PMC7157730

